# Road to The Red Carpet of Edible Crickets through Integration into the Human Food Chain with Biofunctions and Sustainability: A Review

**DOI:** 10.3390/ijms23031801

**Published:** 2022-02-04

**Authors:** Varongsiri Kemsawasd, Woorawee Inthachat, Uthaiwan Suttisansanee, Piya Temviriyanukul

**Affiliations:** Food and Nutrition Academic and Research Cluster, Institute of Nutrition, Mahidol University, Salaya, Phuttamonthon, Nakhon Pathom 73170, Thailand; varongsiri.kem@mahidol.ac.th (V.K.); woorawee.int@mahidol.ac.th (W.I.); uthaiwan.sut@mahidol.ac.th (U.S.)

**Keywords:** *Acheta domesticus*, Alternative food, bioactivity, crickets, edible insects, food security, *Gryllus bimaculatus*, sustainability

## Abstract

The Food and Agriculture Organization of the United Nations (FAO) estimates that more than 500 million people, especially in Asia and Africa, are suffering from malnutrition. Recently, livestock farming has increased to supply high-quality protein, with consequent impact on the global environment. Alternative food sources with high nutritive values that can substitute livestock demands are urgently required. Recently, edible crickets have been promoted by the FAO to ameliorate the food crisis. In this review, the distribution, nutritive values, health-promoting properties (antioxidant, anti-inflammatory, anti-diabetic and anti-obesity), safety, allergenicity as well as the potential hazards and risks for human consumption are summarized. Cricket farming may help to realize the United Nations sustainable development goal No. 2 Zero Hunger. The sustainability of cricket farming is also discussed in comparison with other livestock. The findings imply that edible crickets are safe for daily intake as a healthy alternative diet due to their high protein content and health-promoting properties. Appropriate use of edible crickets in the food and nutraceutical industries represents a global business potential. However, people who are allergic to shellfish should pay attention on cricket allergy. Thus, the objective of this review was to present in-depth and up-to-date information on edible crickets to advocate and enhance public perception of cricket-based food.

## 1. Introduction

By 2050, the world population is predicted to expand to 9.7 billion people, resulting in a two-fold increase in food production [1,2]. However, due to current severe climate change, large rates of food production may not be possible as in the past. Climate change also makes predicting food quantities each year more challenging. Therefore, increased population will speed up food insecurity in the future [3]. To solve the problem, alternative food sources that can substitute livestock demands must be instantly considered. The five requirements should be met by the proposed alternative food, including (i) high nutrient contents, (ii) health-promoting properties, (iii) no or low adverse effects (iv) high sustainability and (v) consumer acceptance. To the best of our knowledge, edible insects are great of interest. Indeed, the Food and Agriculture Organization of the United Nations (FAO) supports entomophagy (the practice of eating insects) by promoting edible insects as a future food source to alleviate food insecurity [4].

Among edible insects, edible crickets are particular favorite for insect eater even in Thailand as it contributes to 31.6% in the global market for edible insects [5]. Furthermore, crickets farming is rapidly growing to supply food industry. Hence, as it gains more attention recently, we focus solely on the edible crickets in this review. Although there are some review articles on edible crickets, most of them described mainly on the nutritive values and sustainability leaving some gap of knowledge. Our review fills the remaining gap of knowledge by covering other parts of crickets, such as distribution of edible crickets, health benefits of edible crickets in a molecular basis and their safety and allergenicity. Thus, this review was then structured into six sections starting with (i) distribution of edible crickets followed by (ii) nutritive values of edible crickets, (iii) health benefits of edible crickets, (iv) safety aspects of crickets, (v) sustainability of edible crickets and (vi) future perspectives.

## 2. Approach

The objective of this review was to present in-depth and up-to-date information on edible crickets as an alternative food source. Scientific literatures, including research articles and books were gathered from various databases using appropriate keywords. The main scientific databases were PubMed, ScienceDirect and Scopus. Beside scientific literatures, cricket legislations and some books provided by international organization, such as FAO were assessed via Google. The combination of keywords (first and second keywords) and obtaining publications from three major databases were shown in Table 1. To provide an up-to-date data, the publication date before 1999 were excluded, and more than 80% of the used scientific materials should have been published since 2012.

**Figure 1:**
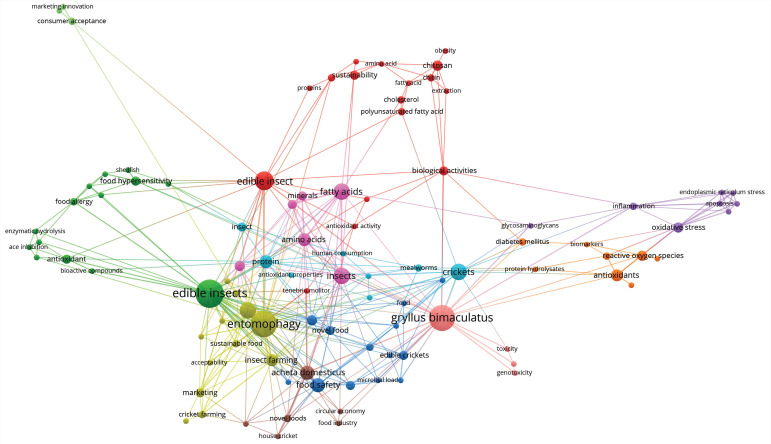
Analysis of co-occurrence links between keywords of used bibliographic sources in this review (counted when each keyword appeared at least twice).

The bibliographic sources used in this review were subsequently analyzed using VOSviewer (version 1.6.17) from Leiden University, The Netherlands [6] for the co-occurrence links between keywords (counted when each keyword appeared at least twice). According to Figure 1, the most relevant keywords (determined by the size of the circles) presented in the used bibliographic resources were edible insects (23 occurrences), entomophagy (21 occurrences), *Gryllus bimaculatus* (20 occurrences), food security (10 occurrences) and crickets (10 occurrences).

## 3. Distribution of Edible Crickets

The history of insect consumption as human food has been widely documented. Half Hours with Insects, published in 1877 informed about the consumption of ants in India, bee pupae in China, grasshoppers in Arabia and Africa, bees and ants in Mexico and Sweden, silkworms and larvae of hawkmoths in China and palm weevils in the West Indies [7,8]. Nowadays, the global perspective of entomophagy is driven by specific conditions and cultures. Countries with warm and moist tropical climates are more renowned for insect rearing than those in temperate zones [4]. Populations in the Asia Pacific region adapted to entomophagy as their culture, and have long acknowledged the nutritional and economical values of edible insects [4,9,10]. Western populations are now showing increased awareness toward the positive impacts of edible insects on the environment and human health [8].

Increasing human interest in entomophagy has led to the development of rearing processes and the export of insect foods, especially crickets [11]. Countries in Australasia and Asia have applied three different techniques for insect rearing including wild harvesting, semi domestication and farming [12]. Insect farming is the most applicable method in many developed countries because it is both cost-effective and profitable [13]. For product marketing, Peru in South America is a renowned producer of cochineal, accounting for 85% of global production [4]. The Central African Republic exports 8 tons of dried mopane caterpillars to Europe every year [4], while HaoCheng Mealworm Inc. (Hunan, China) in China exports 200 tons of dried mealworms annually [4]. In developing countries in Southeast Asia, insect rearing in rural areas is common in Thailand, Cambodia, Vietnam and the Lao People’s Democratic Republic (Lao PDR). In 2013, Thailand, a renowned country for edible insects, had an annual production of 7000 tons of crickets from over 20,000 cricket farmers [11,14]. Safety evaluation must be conducted before product marketing in some countries because edible insects are regarded as a novel food under EU regulation 2015/2283 for European countries [15] and the Niger’s Décret No. 2011-616/PRN/MEL Republic of the Niger in Africa [16].

The *Coleoptera*, *Lepidoptera*, *Hymenoptera*, *Orthoptera*, *Hemiptera*, *Isoptera*, *Odonate*, *Diptera*, *Dictyoptera* and *Megaloptera* are taxonomic orders of global edible insects, with the most renowned as *Coleoptera* or the beetles [4]. When considering nutritional value, cricket species in the *Gryllidae* family, *Orthoptera* are a high protein-rich source [17,18] and popular spotlights of the food industry [10]. In food product development, species of edible crickets generally comply with rearing standards as short development cycle, low feed conversion ratio, high survival rate, high ability to live and high resistance to disease [4]. 

**Figure 2:**
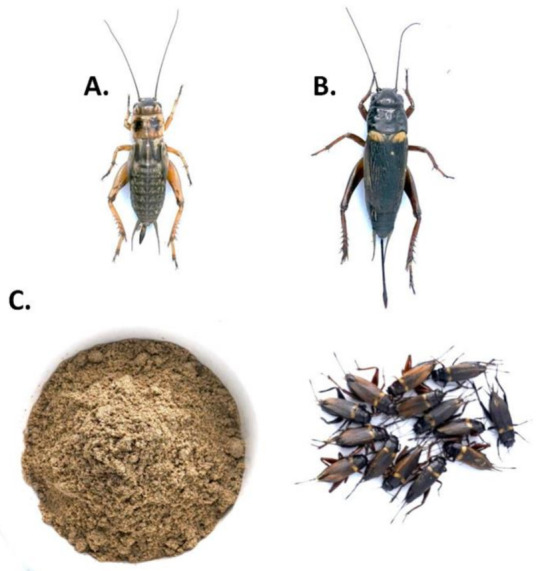
A representative image of (**A**) *Acheta domesticus*, (**B**) *Gryllus bimaculatus* and (**C**) Cricket powder made from *G. bimaculatus*.

Over 2400 species of crickets have been globally identified, and almost half belong to the *Gryllidae* family [19]. The *Gryllidae* contains 62 popular edible species, mostly distributed in Asia (41 species) and Africa (26 species), while 5 species are found in America, 4 in Europe and 4 in Australia [14]. *Brachytrupes portentosus* is the largest cricket in the family of *Gryllidae* [20], with availability limited to Africa [14]. Most reared cricket species for consumption is *Acheta domesticus* [20]. *Gryllus bimaculatus* is mostly reared in Asia, Africa and Australia, while *A. domesticus* is reared on all continents except Antarctica [14]. Favorable conditions for crickets are warm temperature (29–35 °C) and high relative humidity (50%) [21]. Figure 2 shows a representative image of *A. domesticus*, *G. bimaculatus* and cricket powder.

Crickets are farmed commercially worldwide, however, the rearing technology varies and depends on many factors, which may affect their development and nutritional profile as for instance rearing environment [22] and diets. Regarding lighting, different light intensity and duration were applied to improve production efficiency [4,23,24]. Various use of rearing materials as conventional (egg carton, cotton wool and plastic bucket) and improvised (bamboo, scrap blanket and plywood-based cage) materials has no effect on their growth [25]. The composition of diet plays crucial role on growth and nutritional quality of crickets [26]. Moreover, nutritive value of edible insect can be tailored by a supplement of dietary sources [27,28]. In Europe, insects are considered as farmed animals, and must be fed using safe diets. Regulations of the European Parliament and the EU Council obligate the use of animal feed and plant materials [29].

To serve Western populations that are not familiar with entomophagy, cricket farms and industries in Western countries formulate various kinds of cricket-based foods in the form of dried cricket and cricket flour as for instance cricket bars [11,30,31]. Many cricket farms and industries are listed in Europe and America. In North America, the first established farms were Armstrong Cricket Farm in 1945 and Ghann’s Cricket Farm in 1952. The number of cricket farms gradually increased including the Entomo Farm [11], Big Cricket Farms, All Things Bugs LLC and Griopro [31]. In the United States, the largest cricket farms in 2013 were Aspire Food Group and All Things Bugs [11]. In Europe, the first cricket farm was established in the Netherlands as Kreka, followed by Entocube in Finland [11] and Crickeatz in the United Kingdom [31]. Europe and North America also have cricket industries including Crickets, Crickets Crackers, Cricket Flours, Cricket Foods, The Cricket Girl, Dimini Cricket and The Gourmet Cricket Co. 

In Thailand, production of edible crickets began in 1998 and over 20,000 cricket farmers are now registered in the country [20]. Initially, cricket rearing only focused on the three native species *G. bimaculatus*, *Teleogryllus mitratus* and *Teleogryllus occipitalis* [20]. However, recently, farmers have shifted their interest into mass rearing of *G. bimaculatus* and *A. domesticus* that offer a shorter production cycle and greater returns [8]. These two species have a development cycle of 45 to 60 days [32], with advantages of *A. domesticus* production outweighing *G. bimaculatus*. *G. bimaculatus* stands out on economic factors (e.g., market value and sustainable development (SDG 12) [15]), while *A. domesticus* has a more acceptable taste (e.g., nutty and flavor of umami) [20]. *Acheta domesticus* also has a better survival rate [25,33], with higher micronutrient and protein contents than *G. bimaculatus* [34,35].

The Food and Agriculture Organization of the United Nations (FAO) reported abundant *A. domesticus* reared in Thailand [4] and exported to Western countries [32]. Thai farmers conventionally rear crickets in their backyards using different types of pens including concrete cylinders, concrete blocks, plywood boxes and plastic drawers with covers to protect from other animals [32]. Initially, farmers fed their crickets with commercial insect nourishment containing 14–21% protein to boost nutritional values [32]. The formulated cricket feed market is constantly growing along with the increasing numbers of cricket farms in Thailand [11]. Shortly before harvest, the crickets are fed with vegetables, vegetable by-products and fruits to improve taste and nutritional profile [4,36]. Nowadays, mass production of cricket flour and cricket snacks is conducted by Thailand Unique and Smile Bull Companies, while Global Bugs is a leader in cricket flour making [11].

In 2017, the National Bureau of Agricultural Commodity and Food Standards, Ministry of Agriculture and Cooperatives, Thailand launched the Thai agricultural standard on Good Agricultural Practices (GAP) for Cricket Farming TAS 8202-2017 [37]. This voluntary standard provided guidelines for the safe practice of cricket rearing. The GAP for cricket farming consists of five subsections related to farm components, farm management, animal health, the environment and record keeping. These requirements mitigate the occurrence of potential hazards and adverse effects of cricket rearing on the environment. The GAP also focuses on waste disposal handling. The Food and Agriculture Organization of the United Nations (FAO) in collaboration with an entomologist from Khon Kaen University, Thailand issued the ‘Guidance on sustainable cricket farming’ that details procedures for sustainable cricket rearing and farm inspections.

## 4. Nutritive Values of Edible Crickets

The FAO recommends edible insect consumption, including crickets, because of their high nutritive values, especially protein and fat [4]. This review summarizes the proximate analysis, protein and fat contents of four edible crickets commonly consumed in Thailand including *A. domesticus*, *B. portentosus* and the two field crickets *G. bimaculatus* and *Gryllus testaceus*. Values in Table 2 demonstrate edible crickets as rich sources of protein, especially *A. domesticus* (64.1–71.7% dry matter (DM)), while *B. portentosus* showed the lowest protein content. However, more research is required on cricket species with limited data. Besides species, the nutritional profiles of cricket were altered by developmental stage and rearing conditions (e.g., feed and temperature) [26]. Interestingly, both field cricket species from different global regions (China, Korea, Thailand) showed similar protein contents ranging from 58.2 to 60.7% DM but more variation in fat content (10.30–23.40% DM). Adult *G. bimaculatus* grown in Thailand exhibited the highest fat content (23.40% DM). *Gryllus bimaculatus* had high protein content compared to egg, chicken, beef, pork, soybean and maize [38,39], while protein content was slightly lower than fish [39]. The fat content of *G. bimaculatus* was, as expected, higher than plant food such as soybean, maize and wheat [39]. An intake of 200 g of *G. bimaculatus* was sufficient to satisfy daily fat energy requirements [40]; however, fat contents of different cricket species varied as shown in Table 2.

Edible crickets contain high protein contents, and detailed investigations have been conducted on their amino acid profiles [34,38,45]. Amino acids can be divided into two groups as essential and non-essential, based on bodily requirements. Essential amino acids cannot be synthesized by the body and must come from food intake, while non-essential amino acids are synthesized in the body. Amino acids play various vital roles. They are a major component of glutathione, the most abundant low molecular weight antioxidant in cells [46], and also act as precursors to neurotransmitters and signaling regulators [47,48]. Protein deficiency or malnutrition leads to osmotic imbalance, resulting in kwashiorkor. Table 3 presents the amino acid compositions of the four edible crickets. The high total protein and essential amino acid contents indicated good quality protein sources. Various amino acid compositions across species suggested that species and other factors (feed, sex and method of measurement) contributed to the diversity. Indeed, Kulma et al. (2019) reported that sex could be an important factor contributing to protein and lipid levels because female *A. domestica* showed lower amount of protein, but higher in lipid compared to male *A. domestica* [49]. Regarding the protein contents, it has been revealed that crickets have chitin presented on their exocuticle and endocuticle. Chitin is a naturally polysaccharide binding to nitrogen, thus protein in crickets calculated as nitrogen content ×6.25 (nitrogen-to-protein conversion factor, k_p_) by Kjeldahl or elemental analysis methods may be overestimated [35,50,51,52,53,54]. According to Boulos et al. (2020), the protein content of whole insects should be calculated using the k_p_ value of 5.33 rather than 6.25 in order to provide a suitable protein content in insects [52]. In addition, Ritvanen et al. (2020) proposed a k_p_ value of 5.00 for *A. domesticus* and *G. bimaculatus* [35]. Hence, in order to minimize overestimation of crude protein owing to chitin bound nitrogen, a precise measurement of insect chitin is required, Han and Heinonen (2021) developed an ultra-high performance liquid chromatographic and fluorescent method (UPLC/FLR) to analyze chitin from insects [54]. After alkaline deproteinization and acid hydrolysis of chitin, the UPLC/FLR method exhibits sensitive and specific quantification of insect chitin [54].

As well as proteins, fatty acids are crucial macromolecules that can be used as dietary energy, organ insulation and cell membrane components. Table 4 shows the fatty acid profiles of four edible crickets, with limited data available for *B. portentosus* and *G. testaceus*. Crickets contain both saturated fatty acids (SFA) and unsaturated fatty acids (UFA). SFAs have been extensively reported for their adverse health effects including increased risk of cardiovascular disease (CVD) [56], while polyunsaturated fatty acids (PUFA) contribute health benefits by reducing plasma cholesterol [57] and increasing high-density lipoprotein (HDL) [58], which is associated with lower risk of CVD. Palmitic acid and stearic acid were the predominant SFAs in crickets, particularly in *A. domesticus* and *G. testaceus*, while PUFA as oleic acid and linoleic acid were mainly presented, especially in *G. testaceus*. Fatty acid profiles revealed that *G. bimaculatus* had lower SFA compared to pork, beef and egg. Conversely, PUFA in *G. bimaculatus* were higher than in pork, beef and egg [38]. Furthermore, *G. testaceus* showed the highest PUFA:SFA ratio (3.480) followed by *B. portentosus* (1.554). Increasing consumption of fat with high PUFA:SFA ratio has been suggested for CVD prevention [59]. The PUFA:SFA ratio in various species animals have been reported as follows, fish (0.50–1.79), chicken (0.93–0.94), pig (0.46–0.48), cattle (0.11–0.20) and lamp (0.13–0.37) [60], implying the high quality of fat components of *G. testaceus* and *B. portentosus*. However, more information is still needed to conclude since several fatty acids are missing. Similar to amino acid profiles, the varied fatty acid compositions implied that species and other factors such as feed, sex and method of measurement contributed to the diversity [61].

## 5. Health Benefits of Edible Crickets

Edible crickets show benefits to humans including antioxidant, anti-inflammatory, anti-diabetic and anti-obesity activities, thereby enhancing the positive effects of consumption. This section summarizes the health-promoting properties of crickets and their bioactive substances on a molecular level.

### 5.1. Antioxidant Activities

Reactive oxygen species (ROS) or free radicals are generated during cell oxidative metabolism in mitochondria. They subsequently interact with nucleotides, lipid and protein resulting in cellular dysfunctions, premature aging and even cancer [62,63]. Actions of ROS are balanced in cells by antioxidative agents and antioxidant enzymes. Fruits and vegetables are abundant in phytochemicals and well-known as sources of antioxidative agents that quench free radicals in vitro and in vivo [64,65,66]. To determine the antioxidant properties in vitro, three assays are generally employed based on their ability to decrease free radicals as oxygen radical absorbance capacity assay (ABTS), 2,2-diphenyl-1-picrylhydrazyl (DPPH) scavenging assay and ferric reducing antioxidant power (FRAP) assay. The ABTS assay relies on the hydrogen atom transfer (HAT) mechanism to quench free radicals, while DPPH and FRAP reduce free radicals via single electron transfer (SET) [67]. To measure antioxidant properties in vivo, expression of several antioxidant enzymes such as catalase, glutathione peroxidase (GPx), superoxide dismutase (SOD) and DNA lesions derived from oxidative damages such as 8-hydroxy-2’-deoxyguanosine (8-OHdG) are generally used as biomarkers.

**Figure 3:**
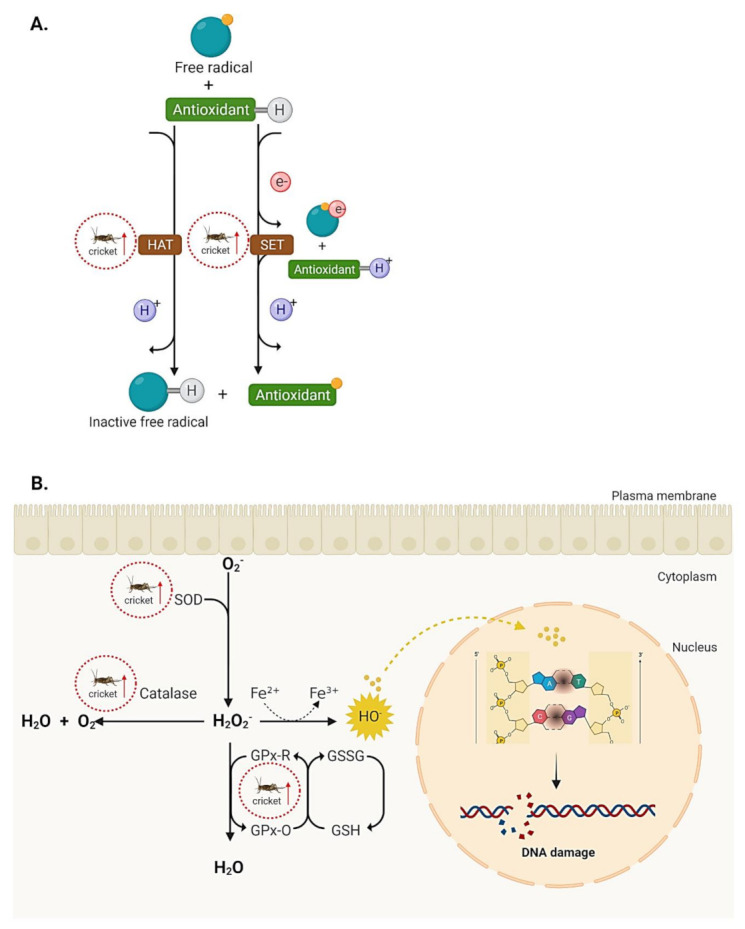
Antioxidant mechanisms showing how crickets exhibit antioxidant properties. (**A**) In vitro antioxidant mechanism through HAT and SET mechanisms. (**B**) Cellular antioxidant mechanisms through catalase, GPx and SOD. Improper regulation of free radicals may lead to DNA damages. GPx: glutathione peroxidase; GSH: glutathione; SOD: superoxide dismutase.

The antioxidant activities of *G. bimaculatus* and *A. domesticus* are summarized in Table 5 and Figure 3; however, no information on *B*. *portentosus* and *G*. *testaceus* is available. Adult *A. domesticus* extracted with ultrasound-assisted extraction (UAE) using absolute ethanol or 50% (*v/v*) aqueous ethanol exhibited antioxidant activities. The aqueous ethanolic fraction with a higher polarity index than absolute ethanol exhibited high antioxidant activities against 2,2-diphenyl-1-picrylhydrazyl (DPPH) radicals at 80–90% DPPH inhibition. This was 2 to 3 folds higher than absolute ethanol, suggesting that the antioxidative agents were water-soluble compounds [68]. However, the polarity index showed reduced influence when crickets were extracted by pressurized liquid extraction (PLE). Extraction by UAE or PLE enriched the crude extracts with several compounds including fatty acids, amino acids, organic acids and carbohydrates [68]. The water-soluble fraction of *A. domesticus* also exhibited antioxidant activity comparable to fresh orange juice, measured by the reduction of ferric iron (Fe^3+^) to ferrous iron (Fe^2+^) through the FRAP assay [69]. Protein hydrolysates prepared from three commercial proteases (Alcalase, Flavourzyme and Protamex) showed DPPH radical scavenging activities with ~2 to 26.5% inhibition using extract concentration of 0.05–5 mg/mL [70]. The protein hydrolysate prepared from Alcalase digestion exhibited the highest inhibition of DPPH radical scavenging activities (26.5%) resulting from the high amount of low molecular weight peptides (LW-MWPs) during hydrolysis. LW-MWPs (1–10 kDa) were shown to be more powerful in quenching oxidants compared to high molecular weight peptides [71].

For antioxidant activities, the ethanolic extract of *G. bimaculatus*, displayed vitamin C equivalent antioxidant capacities against DPPH radicals at 25.4 mg/g sample [72], while cricket protein isolate (CPI) from *G. bimaculatus* exhibited DPPH scavenging activity in a dose-dependent manner [73]. Interestingly, the ethanolic extract of *G. bimaculatus* reduced lipopolysaccharides (LPS) or palmitate-induced ROS production in macrophages measured by the DCFDA assay [74]. The ethanolic extract also prevented alcohol-mediated 8-OHdG formation in mouse intestine [72]. The 8-OHdG is a biological marker resulting from guanine-attacked ROS [75]. To control ROS and 8-OHdG production in the body, several antioxidant enzymes including catalase, GPx and SOD act in concert to reduce ROS. *G. bimaculatus* powder also increased testicular catalase, GPx and SOD in varicocele-induced rats, leading to decreased ROS [76].

**Table 5:** Antioxidant activities of *A. domesticus* and *G. bimaculatus* determined by various readouts.

Species	Extraction Methods	Antioxidant Methods	Antioxidant Activities	Ref.
*A. domesticus*	ultrasound-assisted extraction	DPPH	+	[68]
	pressurized liquid extraction	DPPH	+	[68]
	enzymatic hydrolysis	DPPH	+	[70]
	aqueous extraction	FRAP	+	[69]
	aqueous extraction	ABTS	+	[69]
	hexane extraction	ABTS	+	[69]
*G. bimaculatus*	ethanolic extraction	DPPH	+	[72]
	aqueous extraction	ABTS	+	[72]
	protein isolate	DPPH	+	[73]
	subcritical water hydrolysates	DPPH	+	[77]
	subcritical water hydrolysates	FRAP	+	[77]
	fermented with probiotics	DPPH	+	[78]
	fermented with probiotics	FRAP	+	[78]
	fermented with probiotics	SOD-like activity	+	[78]
	aqueous extraction	Prevention of 8-OHdG formation	+	[72]
	ethanolic extraction	Prevention of 8-OHdG formation	+	[79]
	ethanolic extraction	DCFDA	+	[74]
	insect powder	Total free radicals	+	[76]
	insect powder	Catalase activity	+	[76]
	insect powder	GPx activity	+	[76]
	insect powder	SOD activity	+	[76]

DPPH: 2,2-diphenyl-1-picrylhydrazyl; FRAP: ferric reducing antioxidant power; ABTS: 2,2’-azino-bis-3-ethylbenzothiazoline-6-sulfonic acid; SOD: superoxide dismutase; 8-OHdG: *8*-hydroxy-2’-deoxyguanosine; DCFDA: 2′,7′-dichlorofluorescin diacetate; GPx: glutathione peroxidase.

### 5.2. Anti-Inflammatory Properties

**Figure 4:**
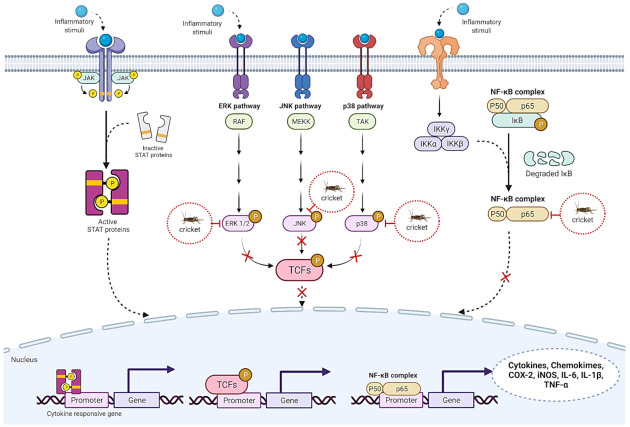
Inflammatory response pathway showing how crickets and their bioactive substances exhibit anti-inflammatory properties. ERK1/2: extracellular signal-regulated kinase 1/2; JNK: c-Jun N-terminal kinase; p38: p38 mitogen-activated protein kinases; TCFs: transcription factors; JAK-STAT: Janus kinase -signal transducer and activator of transcription; NF-κB: nuclear factor kappa B.

Inflammation is a biological process responding to microbial infection or tissue damage. During inflammation, immune cells are activated and release several signaling molecules and cytokines including prostaglandin, interleukin (IL), tumor necrosis factor alpha (TNF-α) and nitric oxide (NO) (Figure 4). The inflammation process is quenched when damages are repaired. Prolonged exposure to these molecules leads to cellular dysfunction and inflammation-related disorders including type II diabetes, cancer, non-alcoholic fatty liver disease, chronic kidney disease, chronic arthritis and neurodegenerative disorders [80]. Hence, reducing chronic inflammation protects normal cell function.

The role of edible crickets in reducing inflammatory response has shown promise. However, only *G. bimaculatus* has been extensively studied both in vitro and in vivo, due to its abundant availability. Treatment of varicocele (VC)-induced male rats with fine powder of *G. bimaculatus* showed that rats receiving *G. bimaculatus* powder exhibited low levels of testicular TNF-α and IL-6 compared to the VC control [76]. VC induction is characterized by enlarged veins inside the scrotum, and tortuosity of the internal spermatic vein leads to testicular inflammation [76] and male infertility [81]. Compared with pure insect powder, the ethanolic extract of *G. bimaculatus* reduced inflammatory cytokines such as TNF-α, IL-1β and IL-6 in macrophage cells (RAW264.7 cells) at both transcriptional and translational levels in a dose-dependent fashion when lipopolysaccharide (LPS) or palmitate was used as an inflammation inducer. Reduction of the inflammatory cytokines occurred by suppression of the nuclear factor kappa-light-chain-enhancer of activated B cells (NF-κB signaling) and mitogen-activated protein kinase (MAPK), which are crucial cell signaling pathways for cytokine production (Figure 4). *G. bimaculatus* decreased inflammatory cytokines by suppression of inflammasome formation [74]. The immunohistological analysis also showed that the ethanolic extract of *G. bimaculatus* inhibited IL-1β level in liver Kupffer cells of mice treated with alcohol [72]. These data suggested that the bioactive compounds combating inflammation were water-soluble such as proteins, peptides or polysaccharides. Cricket protein decreased nitric oxide (NO) production in LPS-treated RAW264.7 cells [82]. Surprisingly, cricket protein hydrolysates prepared by food grade protease digestion (Flavourzyme, Alcalase and the mixture between them) resulted in low molecular weight proteins (below 15 kDa) that did not show anti-inflammatory activity regarding LPS-mediated NO production in RAW264.7 cells [82]. The authors hypothesized that bioactive peptides harboring anti-inflammatory activities may be degraded and inactivated during enzymatic hydrolysis [82]. Further investigation on the anti-inflammatory activities of protein hydrolysates by measuring other inflammatory markers is necessary to confirm these findings. Interestingly, glycosaminoglycans, a type of linear polysaccharides, isolated from *G. bimaculatus* exerted anti-inflammatory properties by (i) reducing LPS-induced prostaglandin E2 production in RAW 264.7 cells, and (ii) reducing the IL-6 level in Freund’s adjuvant (CFA)-induced rat paw edema [83]. Thus, *G. bimaculatus* and its functional components (bioactive proteins, peptides and polysaccharides) exhibited anti-inflammatory properties in vitro and in animal models, implying that *G. bimaculatus* could be used as an alternative functional food to ameliorate various inflammation-related disorders.

### 5.3. Anti-Diabetic Properties

Diabetes mellitus is a chronic disease characterized by insulin deficiency leading to high blood sugar levels (hyperglycemia) [84]. The global estimate of diabetes prevalence in 2019 was 463 million diabetes mellitus suffers. This figure is forecast to increase to 548 and 700 million by 2030 and 2045, respectively [85]. There is no cure for diabetes mellitus and the current therapeutic strategy is to control blood sugar levels. Diabetes mellitus has a high impact on individuals, families and global society. There are many types of anti-diabetic drugs including (i) α-glucosidase inhibitors, (ii) insulin sensitizers and (iii) hepatic gluconeogenesis inhibitors. However, anti-diabetic properties were only reported in *G. bimaculatus* performed exclusively using raw samples harvested in Korea [82,86]. This review summarizes the anti-diabetic properties of edible crickets ranging from fine cricket powder to isolated glycosaminoglycan.

Streptozotocin-induced type I diabetic rats were treated with finely powdered *G. bimaculatus* and blood glucose measurements and insulin tolerance tests were investigated. Data showed that diabetic rats receiving *G. bimaculatus* (1.63–6.5 g/kg) rescued glucose and insulin tolerance in a dose-dependent fashion [86]. Immunoblotting for detection of proteins involved in apoptosis and protein kinase B (AKT)/mammalian target of rapamycin (mTOR) pathway associating with β cells mass and insulin resistance [87] revealed that *G. bimaculatus* prevented streptozotocin-induced pancreatic damage, while enhancing AKT and mTOR Complex 1 (mTORC1) signaling involving phosphorylated form of mTOR, 70-kDa ribosomal protein S6 kinase beta-1 (p70S6K) and 4E-binding protein 1 (4EBP1) in the pancreas. These results suggested that *G. bimaculatus* protected pancreatic β-cell function toward a diabetic state through prevention of apoptosis and enhanced AKT/mTOR signaling [86]. *G. bimaculatus* powder digested with a single protease (Flavourzyme or Alcalase) to generate protein hydrolysate exhibited low anti-α-glucosidase activity (~5–10% inhibition at 0.5–1.0 mg/mL protein concentration), while activity significantly increased (34–35% inhibition at 2.0 mg/mL protein concentration) when the combination of two proteases was employed [82]. Protein hydrolysates prepared from the combination of Flavourzyme and Alcalase resulted in smaller protein peptides (below 15 kDa), with specific amino acid sequences that acted as effective α-glucosidase inhibitors [82]. In support, *G. bimaculatus* fermented with probiotics, *Lactobacillus plantarum* MKHA15, resulted in smaller sized peptides that displayed anti-α-glucosidase activity in a dose-dependent manner (>96% inhibition at 30 mg/mL protein concentration), while the control (non-fermented) showed extremely low anti-α-glucosidase activity, even at 50 mg/mL (approximately 10%) [78]. Glycosaminoglycan isolated from *G. bimaculatus* was also treated in type II diabetic mice models (BKS.Cg-m+/+Lepr^db^, heterozygous (db/+) (DB-Hetero, normal) and homozygotes (db/db) (DB-Homo, diabetes). Results showed that after one week of glycosaminoglycan treatment blood glucose levels reduced, while diabetic liver was ameliorated [88]. In summary, *G. bimaculatus,* especially after digestion, possessed anti-diabetic properties in vitro and in vivo with various underlying mechanisms including α-glucosidase inhibitory activity, pancreatic β-cell function maintaining and diabetic liver amelioration.

### 5.4. Anti-Obesity Properties

**Figure 5:**
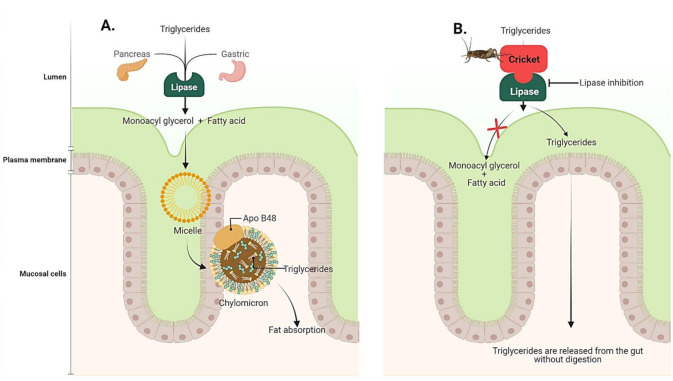
Digestion and absorption of triglycerides by lipase. (**A**) Dietary triglycerides are digested by lipase, which will be subsequently absorbed. (**B**) Lipase inhibition by cricket extract resulted in no absorption of triglycerides.

Obesity is a condition of excessive body fat accumulation that presents a prominent risk for non-communicable diseases (NCDs) including type II diabetes, cancer and hypertension. The recommended approach to reduce obesity is based on reduction of energy intake and exercise; however, this strategy is not effective in the long term [89]. Hence, suppression of dietary fat absorption by inhibition of gastric and pancreatic lipase or reduced food appetite are the core modes of action of anti-obesity agents (Figure 5) [90].

Adult *A. domesticus* extracted with pressurized liquid extraction (PLE) using absolute ethanol or 50% (*v*/*v*) aqueous ethanol gave half inhibitory concentration (IC_50_) against pancreatic lipase in vitro at 0.26 mg extract/mL using pressurized liquid extraction with aqueous ethanol, while IC_50_ value significantly increased to 0.70 mg extract/mL using absolute ethanolic extraction [68]. These results indicated that the bioactive compounds might be water-soluble with moderate to high polarity indices. A naturally occurring compound in insects, as well as in crabs and shrimps, is a polysaccharide called chitin, which can be converted to chitosan [91]. Chitin is insoluble in water and organic solvents [91]. Chitosan isolated from *A. domesticus* trapped lipid, with lipid-binding capacity of 168.7–210.8 g oil per g chitosan, comparable to shrimp chitosan [92], which exerted anti-obesity by controlling bodyweight in an animal model [93].

*G. bimaculatus* extracted with ethanol also displayed anti-obesity properties in a high fat-treated rat model [94]. Rats receiving the extract at 200 mg/kg for two months showed reduced fat weight, particularly abdominal fat [94]. However, whether the extract decreased bodyweight, total cholesterol, total triglyceride, LDL and HDL remained unclear. Rats treated with isolated glycosaminoglycan from *G. bimaculatus* (5 and 10 mg/kg) for one month showed reduction of adipose tissue weight, abdominal fat and epididymal fat [95], suggesting that glycosaminoglycan increased unsaturated fatty acids in adipose tissue, leading to weight loss. Another study showed that the ethanolic extract of *G. bimaculatus* decreased bodyweight, intestinal adipose tissue and total cholesterol in mice exposed to high-fat food for 14 weeks [96]. Interestingly, the influence of cricket extract on obesity was also evident in genes involved in lipogenesis and lipid accumulation, including leptin, adiponectin, acetyl-CoA carboxylase (ACC), fat-specific protein 27 (Fsp27) and peroxisome proliferator-activated receptor gamma (PPAR-γ), which were downregulated [96].

### 5.5. Other Biological Activities

Results suggested that edible crickets may be beneficial to human health. The ethanolic extract of *G. bimaculatus* exerted anti-aging properties by significantly reducing 8-hydroxy-2’-deoxyguanosine (8-OHdG), which is a potential biomarker for oxidative stress and also modulated some genes contributing to stress, metabolism and biosynthesis [79].

The impact of gut microbiota on human health is now better understood, with gut microbiota known to be associated with pathogenesis [97]. Twenty healthy adults aged 18–65 consumed whole cricket powder (25 g/day) for 14 days. Results showed that the probiotic *Bifidobacterium animalis* increased by 5.7 folds, while *Lactobacillus reuteri* decreased by 3 to 4 folds [98]. Chitin from crickets may act as an antimicrobial agent targeting *L. reuteri* [98]. *Bifidobacterium animalis* also showed potential for obesity management by improving anthropometric adiposity biomarkers, especially in abdominally obese women [99], confirming that edible crickets can be used as a functional food to combat obesity.

**Figure 6:**
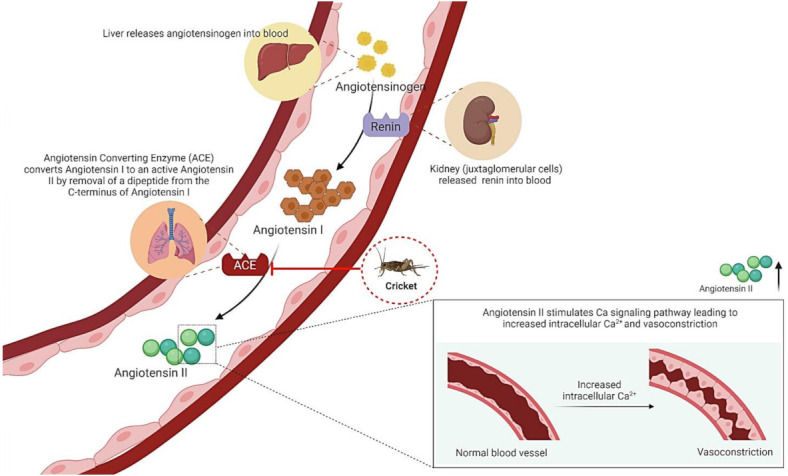
Blood pressure regulation and how crickets extract reduces blood pressure. The angiotensin-converting enzyme (ACE) will convert angiotensin I to angiotensin II, which leads to increased intracellular Ca^2+,^ vasoconstriction and hypertension. Cricket extract inhibits ACE and decrease angiotensin II.

Hypertension and high blood pressure are the most important modifiable risk factors for morbidity and mortality [100]. Blood pressure is controlled through the renin-angiotensin-aldosterone system through inhibition of the angiotensin-converting enzyme (ACE) that converts angiotensin I to angiotensin II as a drug target for hypertensive control (Figure 6). *G. bimaculatus* protein hydrolysate prepared by protease digestion (Flavourzyme, Alcalase and the mixture between them) exhibited high ACE inhibitory activity (~80% inhibition at 0.2 mg/mL protein concentration) in a dose-dependent manner in vitro [82]. Undigested *G. bimaculatus* showed lower ACE inhibition of ~50% using the same extract concentration [82], implying that small peptides derived from protease digestion may enhance ACE inhibition better than non-digested proteins. Hypertension and aging are complex biological processes involving several cellular pathways [100,101]. Further research is required to elucidate the therapeutic properties of crickets; however, current knowledge supports benefits as anti-hypertension and anti-aging.

## 6. Safety Aspects of Crickets

To promote cricket consumption, knowledge of nutritive values, health benefits and safety procedures are paramount. *Acheta domesticus* reared in a closed farming system have been considered safe by the EFSA panel [102] and are used as a food ingredient in several food products such as crackers, breadsticks, meat imitates and snacks. *Acheta domesticus* showed no cytotoxicity up to 250 µg/mL in the three human cell types HL60, HeLa and Caco-2) [102]. Even though no report on toxicity and genotoxicity of *A. domesticus* is reported [102], EFSA panel raised concerns on biological and chemical hazards [103].

The toxicity and genotoxicity of *G. bimaculatus* have been intensively investigated. Oral toxicity testing using a repeated dose of the ethanolic extract of *G. bimaculatus* in rats for 28 days was considered safe, with no-observed-adverse-effect level (NOAEL) at 1000 mg/kg/day according to the Organization for Economic Cooperation and Development (OECD) test guidelines No. 407 [104]. The safety of *G. bimaculatus* was also confirmed by conducting an oral toxicity test using a repeated dose of *G. bimaculatus* powder in rats for 90 days. Results showed a NOAEL value at 5000 mg/kg/day with no histopathological changes [105]. From these data, the acceptable daily intake (ADI) of *G. bimaculatus* powder should be limited to 3000 mg/day, suggesting that *G. bimaculatus* is considered safe for human consumption. As mentioned earlier, glycosaminoglycan isolated from field crickets possesses health benefits, and was also subjected to toxicity testing using a repeated dose for 28 days to determine oral toxicity [106]. Results showed that the NOAEL value for glycosaminoglycan isolated from field crickets was 160 mg/kg/day, which was the highest dose used in the experiment [106]. Beside sub-chronic or chronic toxicity testing, genotoxicity testing must also be employed and used as an indicator to detect mutagens and carcinogens. Genotoxicity testing determined by the Ames test in *Salmonella typhimurium*, the chromosome aberration test in Chinese hamster ovary (CHO) cells and the micronucleus test in young male mice revealed that phosphate-buffered saline (PBS) extracted *G. bimaculatus* showed no induction DNA damages in vitro and in vivo [107]. Information on genome safety of glycosaminoglycan isolated from field crickets has also been reported [106]. Thus, all the evidence indicated the safe consumption of *A. domesticus* and *G. bimaculatus*. However, safety information for *B. portentosus* and *G. testaceus* is still lacking.

**Figure 7:**
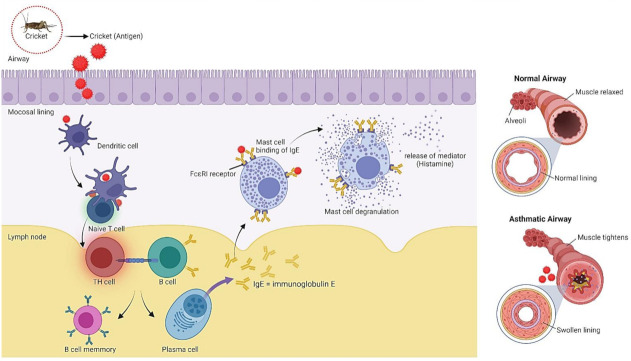
IgE-mediated hypersensitivity by crickets. Dendritic cells recognize cricket antigens, which subsequently lead to the activation of adaptive immune system. Repeated exposure to cricket antigens activates mast cells, resulting in the production of histamine and tightened smooth muscles.

Type I hypersensitivity (IgE-mediated hypersensitivity) including food allergy is associated with allergen-mediated histamine released from mast cells, leading to a red rash, face swelling and in severe cases, anaphylactic shock (Figure 7) [108]. Many food allergies cases are sensitive to arthropods, such as shrimp and crab [109]. Edible insects, including crickets are classified as arthropods; thereby, possessing potential allergenicity. Some cases were reported for insect allergenicity in China and Thailand [110,111], together with cross-reactivity between *A. domesticus*, shrimp and house dust mite [112]. The IgE from patients allergic to shrimp or house dust mite can recognize *A. domesticus* protein, confirming the potential allergenicity of crickets. The cross-reactivity of *G. bimaculatus* and shrimp has also been demonstrated [113]. Hence, crickets appear to be devoid of toxicity but their allergenicity should be considered, particularly for individuals allergic to shrimp and mite.

Crickets have also been tested for potential biological, chemical and physical hazards. Possible human hazards for both raw and processed crickets are summarized below to better control the quality and safety of cricket-based foods.

Raw crickets: Crickets, as a nutrient-rich source, are vulnerable to biological hazards including microbial loads [114], pathogenic bacteria (*Salmonella* sp. and *Shigella* sp.) [115], spore-forming bacteria (*Bacillus, Paenibacillus* and *Psychrobacillus*) [116], high yeast and mold counts (*Debaryomycess*) [20], fungi (*Aspergillus flavus*, *Aspergillus sydowii* and *Cladosporium sphaerospermum*) [117] and parasites (*Balantidium* sp. and *Cestoda* sp.) [118]. Crickets are also susceptible to virus infection such as cricket densovirus (AdDV) and cricket paralysis virus (CrPV); however, these viruses are unlikely threats to human health [103]. Biological hazards in crickets such as viruses, fungi and parasites are categorized as low risk for human health because crickets have the ability to self-detoxify mycotoxin from fungi during the rearing process. These microorganisms can also be reduced through appropriate product processing using heat treatment [117,119,120,121]. However, spore-forming bacteria are categorized as medium risk since they can re-occur after the processing stage [103].

Processed crickets: Biological hazards, especially pathogenic microorganisms in processed crickets rarely occur, except for fungi that can pose a risk during the manufacturing process [103]. Unfortunately, there are no standardized harvest and processing protocols in the US for cricket powder production. High variation of microbial loads in cricket powder occur for aerobic mesophilic counts and fungi as well as for some pathogens, including *Staphylococcus* and *Bacillus*. For example, aerobic mesophilic counts varied from 1.0 to 6.0 log colony forming unit (CFU)/g [122]. Another study on processed crickets focused on the bacterial count of dried, deep-fried and extruded products. Findings highlighted a higher possibility of bacterial reoccurrence in dried crickets than in deep-fried and extruded crickets [123]. *Bacillus* sp., a spore-forming pathogen, is frequently observed in cricket-based foods. The bacilli are strongly related to human diseases including diarrhea and emesis when the value is >6.0 log CFU/g [124]. Some previous studies reported a positive result for *Bacillus* sp. in processed crickets. A study on commercial cricket flour highlighted the presumptive sample load of *Bacillus cereus*, with *Bacillus* count between 1.0 and 3.5 log CFU/g [122]. High prevalence of *Bacillus cereus* in smoked and dried crickets and extruded cricket products has also been reported [45,125].

Thermal treatments such as blanching, boiling, pre-frying, drying and extrusion methods are all used in cricket production. Klunder et al. (2016) reported that both boiling and stir-frying effectively decreased Enterobacteriaceae and bacterial spores [121]. Gatheru et al. (2019) highlighted the effectiveness of freeze-drying and deep-frying methods to eliminate microbes and fungi, while boiling and sun-drying methods only decreased microbial and fungal counts [126]. Vandeweyer et al. (2018) successfully reduced the numbers of microorganisms and spores using various thermal treatments such as freezing, boiling, oven-drying and smoke-drying. They highlighted freezing and boiling as the most effective treatments [125]. Grabowski et al. (2017) varied the cooking time, drying temperature and drying time. They reported a decrease in microbial counts after sequential drying at 80 and 100 °C for 12 h after 30 min of cooking [124].

Microbial counts after 6 h storage at ambient temperature for dried cricket products and frozen cricket products at −25 °C remained the same [125]. By contrast, boiled cricket products at ambient temperature were more susceptible to biological hazards than at chilling temperature (5 to 7 °C) over six months of storage [121]. Results showed a correlation between heat treatment and storage conditions to maintain the safety of edible cricket products. Post-contamination was also related to packaging, labeling and storing processes. Thus, appropriate conditions including the packaging materials, labeling procedures and storage conditions should be monitored to avoid recontamination of fungi and pathogenic bacteria such as *Aspergillus* sp., *Penicillium*, *Fusarium*, *Listeria monocytogenes* and *Salmonella* [120].

Environmental contamination from heavy metals, flame retardants, dioxins and pesticides can also be potential chemical hazards in edible insects. The ‘Guidance on sustainable cricket farming’ highlights the risk of chemical contaminants during the rearing processes. Chemical hazards pertaining to raw and processed crickets include heavy metals (cadmium, arsenic, chromium, lead and tin) [103], allergens (tropomyosin, arginine kinase and the specific allergen in *G. bimaculatus,* hexamerin B1) [103,127,128], polybrominated diphenyl ether (PBDE) flame retardants [129], dioxins (polychlorinated dibenzodioxins and dioxin-like polychlorinated biphenyls) [15] and high pesticide residues (carbamate and organophosphate) [130]. Whole crickets, cricket powder and cricket protein hydrolysates contain tropomyosin; thus, individuals with prawn allergy may experience an allergic reaction after consuming crickets [128]. Cassi et al. (2018) stated that the chemical hazards in crickets, especially allergens and heavy metals, were medium risk because allergen modulation relies on the processing stage, while cadmium also showed high chemical activity [103].

Past decades have seen increasing numbers of reports on microplastic contamination in the soil ecosystem as the living environment of crickets [131,132,133]. Thus, microplastic contamination could be considered a potential physical hazard for crickets. Chemical hazards found in crickets included PBDE flame retardant, which is generally used in consumer plastic products. Gaylor et al. (2011) reported that the accumulation of PBDE in crickets might be sourced from furniture and the automotive sector, where these plastic-based materials commonly accumulate in the terrestrial area [129]. However, the only study on microplastic analysis of natural living insects was conducted in freshwater insects [134].

The occurrence of biological and chemical hazards in crickets, excluding allergens, is mostly caused by unhygienic rearing and production processes [15,103,120]. Therefore, preventive measures should be applied to maintain the safety and quality of cricket products [15] such as Good Farming Practice (GFP), Good Manufacturing Practice (GMP) and Hazard Analysis Critical Control Points (HACCP). Hazard preventive control should include the use of hygiene equipment and safe nourishment for feeding, application of personal hygiene practice, good waste management and good hygiene throughout the food supply chain [120].

Global interest in entomophagy has recently increased. National and regional bodies concerned with food safety in some countries have announced standards for farming and marketing edible crickets. The relevant authorities in some countries now guarantee the quality of edible crickets such as the European Commission (EC) and European Food Safety Authority (EFSA), the Kenya Bureau of Standards (KEBS), the United States Food and Drug Administration (USFDA), the Canadian Food Inspection Agency (CFIA), Food Standards Australia New Zealand (FSANZ) and the Committee on Novel Foods (ACNF) in Australia and New Zealand, the Guangxi Zhuang Autonomous Region in China and the Thai Food and Drug Administration (Thai FDA) and Agricultural Commodity and Food Standards (ACFS) in Thailand [15].

## 7. Sustainability of Edible Crickets

### 7.1. Reasons for Entomophagy

The United Nations (UN) has highlighted a global population of 9.7 billion by 2050, with largest increases in Africa and Asia. Population growth, urbanization and rising incomes are positively correlated with the consumption of animal-based protein as livestock products [2]. Therefore, population growth increases the demand for meat, which is expected to rise 75% between 2017 and 2050 [135]. Protein consumption in developed countries is higher than in developing countries. From 1961 to 2011, protein consumption in developing countries increased by about 25 g/capita/day, whereas the rate was only 12 g/capita/day in developed countries [136]. This growth rate was influenced by human perception of the high nutritional value of livestock products [2]. To combat the increased demand for animal protein, research into alternative protein sources has increased. Entomophagy is now viewed positively as an environmentally friendly process that can benefit the economy and provide sustained nourishment.

### 7.2. Environmental Aspects

Insect rearing has a low impact on the environment [4]. Global warming potential, reported as CO_2_ emission per kg of edible protein, had the lowest results for *A. domesticus* (4.35 kg of CO_2_-eq) [137] and mealworm (14 kg of CO_2_-eq), while the highest was recorded for beef cattle (75–170 kg of CO_2_-eq) [138] (Table 6). Oonincx et al. (2010) conducted small-scale research and reported that the production of CO_2_ by crickets at 1.57 CO_2_ eq (g/kg mass gain) was lower than pig and beef cattle at 80–1130 and 2850 CO_2_ eq (g/kg mass gain), respectively [139]. The low feed conversion ratio and high edible portion of insects also indicate effective utilization for consumption purposes. The feed conversion ratio (net feed consumption of livestock per unit weight gain) of crickets was 1.7, while livestock was above 2.5, with the highest rate for beef cattle at 10 [140]. The edible portion of crickets is high (80%) compared to chicken, pig and beef cattle (55%, 55% and 40%, respectively) [140]. The water footprint as the amount of direct and indirect water used during production was low (4325–5988 m^3^/t) for insect, pig and chicken farming, while the water footprint of beef cattle was significantly higher (15,415 m^3^/t) [141]. The land area required per kg of insect protein was also 50–90% lower than needed for livestock [142].

The 2021 United Nations World Water Development Report states that the withdrawal of global water has increased by 1% per year since 1980 due to the increase in population growth and shifting consumption patterns into livestock meats [143]. Recently, urbanization and rising incomes have led to higher consumption of conventional livestock. Expansion of agricultural land is necessary to satisfy the progressive global demand for livestock. Consequently, 69% of global water withdrawal can be correlated to the agricultural sector [143], with demand estimated to rise by 60% by 2025 [144]. Apart from water withdrawal, 70% of agricultural land is being used for livestock [145]. The growing water demand outweighs its supply, and this increases the risk of water scarcity. From the 1900s to the 2000s, the number of people suffering from water scarcity increased by 58% [146]. Water scarcity occurs in Northeastern China, India, Pakistan, the Middle East and North Africa [147]. By 2050, sharp increases in population growth in Africa (1.3 billion) and Asia (0.75 billion) will exacerbate the rising demand for water availability and also the need for water quality, with populations on both continents suffering from water pollution, mostly caused by agrochemicals [143].

Mass production of edible insects is being widely developed in some countries to reap the outstanding benefits of entomophagy. Extensive production is expected to achieve environmental sustainability and was monitored using the life cycle assessment (LCA), which is a tool for assessing the environmental impact of edible insect production. Climate change, feed conversion, freshwater and land use and human and livestock well-being all focus on the sustainability and environmental aspects [148]. First, the species and life stage of insects should be selected using knowledge on the impact of other life stages [148]. Second, the determination of suitable diet for insects should focus on sustainable feed production such as the use of feed crops to increase the health of local native species for biodiversity enhancement [148] and impact the sustainability value the edible crickets [149]. Even though the utilization of waste is labeled as environmentally friendly, According to Commission Regulation (EC) No 178/2002, Regulation (EC) No 767/2009, the Regulation (EC) No 1069/20097, European union forbid the use of manure and catering waste, especially foodstuffs containing fish and meat to be used as feed for edible insects [29,150,151,152]. Third, greenhouse gas emissions from the rearing process including the excreta of insects, husbandry, housing and feeding should be wisely managed [148]. Fourth, the high nutritional value of insects requires fewer resources [148]. Insect rearing is more environmentally friendly, and requires 7 times less feed, 50 times less water and emits 100 times less greenhouse gas than beef cattle farming [142]. The Global warming potential on the production of *A. domesticus* are 4.5 times less than broiler production [153]. Insect rearing should be performed ethically, with consideration of lowering adverse effects on insect health [148], under conditions similar to the natural environment. For example, housing design should minimize insect stress to reduce the necessity of medicinal usage [31].

Appropriate sustainable environmentally friendly waste management for cricket and beef cattle farms must also be considered. For cricket farming, these processes include the management of cricket feces, food scraps and water effluent. Farmers commonly use cricket feces as organic fertilizer for soil, food scraps as compost for plants and water effluent for irrigating crops after waste sedimentation. Similarly, beef cattle farming utilizes solid waste as manure and compost, while treated liquid waste is recycled as water supply for feedlots [154]. Large-scale beef feedlot industries in Australia, America and Europe apply advanced technology such as anaerobic digestion, thermal treatment and diet modification to maximize the utilization of manure for generating power and extracting nutrients [154].

### 7.3. Economics and Health

Demand for edible insects impacts the livelihood and economy of society, while the development of insect supply chains will create new job opportunities [15,155]. Economical insect farming increases productivity and income, while production is possible throughout the year. In Europe, the expanding attention being paid to edible insect farming is expected to increase the number of direct and indirect jobs in the food supply chain by 2025. Halloran et al. (2017) considered cricket farming to be a livelihood strategy that plays a crucial role in household income and social and human capital [155]. *A. domesticus* can be harvested multiple times throughout the year [32]. In Thailand, household income in rural areas has increased as a result of insect rearing [15,137].

Concerning the health impacts of entomophagy, insects generally have a lower risk of transmitting zoonotic infections than livestock due to the distant taxonomy of insects with humans. Consequently, consumption of edible insects is less harmful to human health than consumption of conventional livestock meats [4]. Zoonotic diseases have been reported to threaten human health through the consumption of conventional livestock meats. For example, avian influenza (H5N1) originated from poultry [156], swine influenza from pigs [157] and anthrax from beef cattle [158]. Another benefit of edible insect products is their nutrient-rich content. They are categorized as nutrient-dense and calorie-dense foods, with the potential to address malnutrition [141,159,160]. Protein contents vary among cricket species.

## 8. Future Perspectives

Globally, insects are processed using various methods before consumption. In Asia, Africa and Latin America entomophagy has been practiced since ancient times and people are familiar with consuming the whole edible insects [161]. Product processing methods include boiling and drying, toasting, frying, steaming, roasting, smoking, stewing and curing [115,161]. In Western countries, North America and Europe, people are not familiar with eating whole insects [161]. Processing methods include pulverizing and grinding to deform the insects into granulated and paste forms [162]. The strategy for promoting entomophagy in Western countries is through granulated and paste forms of insects that are more suitable to produce snack products [161,163]. The EFSA announced the additional step of removing the legs and wings, especially on crickets before consumption; however, this is still debated due to the adverse effect on nutritional properties and lack of scientific evidence [148].

Apart from conventional methods, advanced technology is being developed by scientists to diversify the market variety of insect products as pastes, sauces, powders, flour, snacks and pasta [11,161,164]. In China, the population consumes fried insects [161]. Similarly, in Africa, insects are commonly processed as smoked, toasted, roasted and fried before consumption [161,165]. Nowadays, a wide range of cricket food applications has become a new food industry as extruded food products (cricket biscuit, cricket protein energy bars, cricket chips, cricket crackers and cricket pasta), diet supplements and food preservatives.

Consumer surveys have progressed in parallel with insect production. A study on insect consumption by children from 6 to 11 years old reported that children are not averse to trying insect products [30]. To increase customer interest in entomophagy, knowledge of the nutritional value of insects should be emphasized [142,166] and instilled from childhood. An analysis of consumer perception on edible insects also reported a positive response by most participants with ages ranging from 18 to over 55 years old, and 77% were willing to try entomophagy [159], while 22% preferred to consume insect-based snacks [159]. Thus, the food industry needs to diversify edible insect products or to incorporate the insects with native ingredients. These could be an effective introduction to increase consumer adoption to edible insect foods. For example, a study on cricket-based biscuits confirmed a high sensory result and revealed the just-about-right color, tasty flavor, pleasant odor and soft texture to suggest market demand [163].

Crickets are a high source of protein and can be used to treat protein-energy malnutrition diseases such as marasmus and kwashiorkor [167]. Moreover, the derived product of crickets as glycosaminoglycan has anti-oxidative and anti-inflammatory properties to treat diabetes [88] and arthritis [83], respectively. A mixture of wheat flour and cricket protein was shown to increase iron solubility and the availability of minerals more than the use of conventional livestock protein (chicken, pig and cattle) [168]. Thus, the addition of crickets into a starch-based diet can positively enhance micronutrients [168]. In addition, hydrolysis of cricket protein can produce novel peptides with bioavailability, for instance antihypertension and antidiabetic or antioxidation which can be used for food and pharmaceutical applications [169,170,171].

The application of cricket lipids is limited to the application in food, as they contain high unsaturated fatty acids [43,44,172]. The cricket lipids can also be potentially used as an alternative source of lecithin for food and pharmaceutical uses since their phospholipid patterns are similar to those of lecithin [172]. In addition to its use as a promising food ingredient, the functionality (i.e., antioxidation) of cricket lipids also attracts interest in scientific point of view [69].

Apart from consumption, insect extract can also be used as an effective food preservative. Chitosan, produced from the deacetylation of chitin in insects is a promising polysaccharide that enhances food safety, shelf life and quality control of food products through its antioxidant and antibacterial effects [173]. Comprehensive research has successfully developed chitosan as a bio-based polymer for food packaging [14]. Chitosan prolongs food shelf life. In the food industry, various methods of food preservation using chitosan are direct coating (dipping, brushing and spraying methods), layer-by-layer assembly and bioactive film packaging [174].

While the future looks bright for edible cricket market, manufacturing process development is still a major challenge in the production and industrialization of insect-based products. Emphasis should also be placed on research highlighting the implementation of technology and process innovation for cricket farming and processing. The process of technology and scientific knowledge transfer will help to facilitate commercial exploitation and the social impact of the use of edible cricket.

## Figures and Tables

**Table 1:** Search strings and number of articles obtained from PubMed, ScienceDirect and Scopus until 2021.

Search No.	First Keyword *	Second Keyword *	PubMed	ScienceDirect	Scopus
1	cricket	entomophagy	34	24	91
2	cricket	nutrition	262	68	186
3	cricket	bioactivity	32	10	17
4	cricket	safety	78	42	144
5	cricket	sustainability	166	72	26

* Two keywords within a single search are linked together using the connector AND.

**Table 2:** Nutritive analysis of four non-processed edible crickets.

Species	Sources	Nutritive Values (% Dry Matter)				Ref.
		Carbohydrate	Fat	Fiber	Protein	
*A. domesticus*	Mexico	2.12 ± 0.3	24.00 ± 0.9	6.20 ± 1.5	64.10 ± 1.2	[41]
*A. domesticus*	Thailand	1.60 ± 0.1	10.40 ± 0.1	4.60 ± 0.2	71.70 ± 0.5	[34]
*A. domesticus*	USA	nr	22.80	19.10	64.38	[42]
*B. portentosus*	Thailand	9.74 ± 0.5	20.60 ± 0.6	11.61 ± 0.2	48.69 ± 0.3	[43]
*G. bimaculatus*	Korea	nr	11.88 ± 0.2	9.53 ± 0.5	58.32 ± 0.3	[38]
*G. bimaculatus*	Thailand	0.10 ± 0.01	23.40 ± 0.1	10.00 ± 0.3	60.70 ± 0.4	[34]
*G. testaceus*	China	nr	10.30 ± 0.3	nr	58.30 ± 0.9	[44]

nr: not reported.

**Table 3:** Amino acid composition (g/100 g dry matter) of four non-processed edible crickets.

Amino Acids	Crickets			
	*A. domesticus*	*G. assimillis*	*G. bimaculatus*	*G. testaceus*
**Essential**				
Histidine	1.72 ± 0.02	1.32	2.50 ± 0.08	1.94 ± 0.01
Isoleucine	2.90 ± 0.10	2.12	2.16 ± 0.04	3.09 ± 0.00
Leucine	3.80 ± 0.14	4.96	3.97 ± 0.05	5.52 ± 0.10
Lysine	3.22 ± 0.08	7.91	2.42 ± 0.01	4.79 ± 0.10
Methionine	0.98 ± 0.03	0.63	0.27 ± 0.01	1.93 ± 0.06
Phenylalanine	2.38 ± 0.00	0.72	1.83 ± 0.01	2.86 ± 0.06
Threonine	1.65 ± 0.05	3.55	2.00 ± 0.04	2.75 ± 0.10
Tryptophan	0.43 ± 0.03	0.95	nr	nr
Valine	4.50 ± 0.03	4.62	3.20 ± 0.03	4.42 ± 0.00
**Non-essential**				
Alanine	3.67 ± 0.05	4.02	5.64 ± 0.01	5.55 ± 0.09
Arginine	3.92 ± 0.05	8.64	3.60 ± 0.04	3.68 ± 0.12
Asparagine	4.61 ± 0.23 *	nr	nr	6.29 ± 0.29
Aspartic acid	nr	3.02	nr	nr
Cystine/cysteine	0.40 ± 0.00	0.74	5.10 ± 0.00	1.01 ± 0.02
Glutamic acid	nr	3.64	6.39 ± 0.07	9.07 ± 0.31
Glutamine	6.45 ± 0.05 **	nr	nr	nr
Glycine	2.60 ± 0.15	2.41	3.32 ± 0.01	3.62 ± 0.11
Proline	3.04 ± 0.03	1.26	1.99 ± 0.01	4.50 ± 0.08
Serine	1.59 ± 0.09	0.61	2.73 ± 0.01	3.72 ± 0.07
Tyrosine	2.71 ± 0.10	5.44	2.73 ± 0.02	3.94 ± 0.02
Total amino acids	50.55	56.56 ^+^	53.83	68.68 ^+^
Ref.	[34]	[55]	[38]	[44]

nr: not reported. *: report as Asparagine + Aspartic acid. **: report as Glutamine + Glutamic acid. ^+^: Calculation was performed in this review.

**Table 4:** Fatty acid profiles (% dry matter) of four non-processed edible crickets.

Fatty Acids	Crickets			
	*A. domesticus*	*B. portentosus*	*G. bimaculatus*	*G. testaceus*
C10:0 Capric acid	0.011 ± 0.00	nr	0.008 ± 0.00	nr
C11:0 Undecylic acid	0.003 ± 0.00	nr	0.004 ± 0.00	nr
C12:0 Lauric acid	0.028 ± 0.00	nr	0.045 ± 0.00	0.540 ± 0.04
C14:0 Myristic acid	0.107 ± 0 00	nd	0.271 ± 0.02	0.390 ± 0.02
C15:0 Pentadecanoic acid	0.009 ± 0.00	nd	0.036 ± 0.00	nr
C16:0 Palmitic acid	5.870 ± 0.31	0.021 ± 0.00	9.260 ± 0.77	10.180 ± 0.20
C17:0 Heptadecanoic acid	0.078 ± 0.00	0.002 ± 0.00	0.101 ± 0.01	nr
C18:0 Stearic acid	1.830 ± 0.09	0.473 ± 0.01	2.730 ± 0.25	2.630 ± 0.09
C20:0 Arachidic acid	0.125 ± 0.01	nd	0.212 ± 0.01	nr
C21:0 Heneicosanoic acid	0.005 ± 0.00	nr	0.008 ± 0.00	nr
C22:0 Behenic acid	0.064 ± 0.01	nr	0.049 ± 0.01	nr
C24:0 Lignoceric acid	0.024 ± 0.01	nr	0.037 ± 0.02	nr
C14:1 Myristoleic acid	0.019 ± 0. 02	nr	0.007 ± 0.00	nr
C16:1 Palmitoleic acid	0.153 ± 0. 01	0.009 ± 0.00	0.295 ± 0.07	3.110 ± 0.10
C18:1n-9 trans Elaidic acid	0.031 ± 0. 00	nr	0.044 ± 0.01	nr
C18:1n-9 cis Oleic acid	3.900 ± 0. 24	nr	9.400 ± 2.20	29.580 ± 0.20
C20:1 cis-11-Eicosenoic acid	0.024 ± 0. 01	nr	0.084 ± 0.05	nr
C22:1n-9 Erucic acid	0.014 ± 0. 01	nr	0.020 ± 0.01	nr
C18:2n-6 trans Linolelaidic acid	nd	0.045 ± 0.01	0.011 ± 0.01	nr
C18:2n-6 cis Linoleic acid	1.170 ± 0.36	nr	1.390 ± 0.73	37.820 ± 0.20
C20:2 cis-11,14-Eicosadienoic acid	0.194 ± 0.04	nr	0.173 ± 0.07	nr
C22:6n-3 Docosahexaenoic acid	nd	nd	0	nr
C18:3n-6 cis Linolenic acid	0.007 ± 0.00	nr	0.062 ± 0.01	10.120 ± 0.10
C18:3n-3 Linolenic acid	0 011 ± 0.00	nd	0.014 ± 0.01	nr
C20:3n-6 cis-8,11,14-Eicosatrienoic acid	0.011 ± 0. 00	0.105 ± 0.00	0.077 ± 0.08	nr
C20:3n-3 cis-11,14,17-Eicosatrienoic acid	0.006 ± 0.00	nr	0	nr
C20:4n-6 Arachidonic acid	nd	0.667 ± 0.00	0.005 ± 0.00	nr
C20:5n-3 cis-5,8,11,14,17-Eicosapentaenoic acid	0.057 ± 0.01	nd	0.070 ± 0.05	nr
Total	13.742 ± 0.76	1.321	24.413 ± 2.77	94.370
SFA (saturated fatty acids)	8.145 ± 0.35	0.496 ± 0.01	12.761 ± 1.07	13.740
MUFA (monounsaturated fatty acids)	4.141 ± 0.25	0.054 ± 0.01	9.850 ± 2.35	32.690
PUFA (polyunsaturated fatty acids)	1.456 ± 0.33	0.771 ± 0.00	1.802 ± 0.53	47.940
PUFA:SFA	0.178	1.554	0.141	3.480
OMEGA 3	0.074 ± 0.01	nr	0.084 ± 0.04	nr
OMEGA 6	1.125 ± 0.35	nr	1.545 ± 0.88	nr
Ref.	[34]	[43]	[34]	[44]

nr: not reported; nd: not detected.

**Table 6:** The comparison of CO_2_ emission per kg edible protein between insect and other conventional livestock.

Name of Animal	Global Warming Potential (kg CO_2_-Equivalent)	Reference
Cricket (*A. domesticus*)	4.35	[137]
Mealworm (*Tenebrio molitor*)	14	[138]
Chicken	18–36	[138]
Pig	21–53	[138]
Beef Cattle	75–170	[138]

## Data Availability

Not applicable.

## Abbreviations

ACE	Angiotensin-converting enzyme
ADI	Acceptable daily intake
CFU	Colony forming unit
DM	% Dry matter
DPPH	2,2-Diphenyl-1-picrylhydrazyl radicals
EFSA	The European Food Safety Authority
FAO	The Food and Agriculture Organization of the United Nations
GFP	Good Farming Practice
GMP	Good Manufacturing Practice
HACCP	Hazard Analysis Critical Control Points
IL	Interleukin
LPS	Lipopolysaccharide
LW-MWP	Low molecular weight peptides
MAPK	Mitogen-activated protein kinase
MUFA	Monounsaturated fatty acids
NCDs	Non-communicable diseases
NF-κB	Kappa-light-chain-enhancer of activated B cells
NO	Nitric oxide
NOAEL	No-observed-adverse-effect level
PUFA	Polyunsaturated fatty acids
ROS	Reactive oxygen species
SFA	Saturated fatty acids
TNF-α	Tumor necrosis factor alpha
UN	The United Nations
